# Improvement of Balance, Motor Aspects, and Activities of Daily Living in Parkinson's Disease after a Sequential Multimodal Aquatic- and Land-Based Intervention Program

**DOI:** 10.1155/2023/2762863

**Published:** 2023-01-09

**Authors:** Dielise Debona Iucksch, Juliana Siega, Giovanna Cristina Leveck, Luize Bueno de Araujo, Tainá Ribas Mélo, Vera Lúcia Israel

**Affiliations:** ^1^Postgraduation Program in Physical Education, Federal University of Paraná, Curitiba 82590-300, Brazil; ^2^Postgraduation Program in Public Health, Federal University of Paraná, Curitiba 80060-240, Brazil; ^3^Public Health, Litoral Campus, Federal University of Paraná, Matinhos 83260-000, Brazil; ^4^Department of Physical Therapy Prevention and Rehabilitation, Federal University of Paraná, Curitiba 80210-132, Brazil

## Abstract

Parkinson's disease (PD) is a neurodegenerative, with heterogeneous clinical conditions and motor changes that reduce functioning. Postural instability is one of the motor aspects of disease progression, with a potential increase in the risk of falls, consequently affecting the activities of daily living (ADL). The objective of this study was to verify the influence of a multimodal intervention program (MIP) sequentially applied in aquatic- (AEs) and land-based environments (LEs) on balance, postural control, motor activities, and ADL in people with PD. It is an interventional clinical study with patients in stages 1 to 4 in the Hoehn and Yahr scale, assessed with Berg Balance Scale (BBS), Mini-Balance Evaluation System Test (Mini-BESTest), Unified Parkinson's Disease Rating Scale (UPDRS) II and III, Dynamic Gait Index (DGI), and quiet stance (QS) analysis in a force platform. The MIP was conducted sequentially with aquatic- (AIs) and land-based interventions (LIs) for 12 weeks each, twice a week, each session lasting 1 hour, and a 12-week interval between interventions. The comparison analysis was made with Friedman ANOVA, and the multiple comparisons with Wilcoxon signed-rank, Bonferroni correction, and effect size (*r*). The sample comprised 18 people with PD (66.83 ± 11.74 years). The AI and the full intervention (FI) had a large effect according to BBS. With Mini-BESTest, the LI and FI had a large effect. According to UPDRS II, the MIP improved ADL after LI, with a medium effect, and the motor aspects of UPDRS III improved after LI and FI, with a large effect. DGI was not sensitive in the analyses, with a ceiling effect after FI. No differences were identified in QS analyses. This research identified improved balance, ADL, and motor aspects in people with PD after sequential MIP in AI and LI, indicating that land-based and aquatic interventions are complementary and advantageous to people with PD.

## 1. Introduction

Parkinson's disease (PD) is the second most common neurodegenerative disease, reported as a motor clinical syndrome, although its clinical condition is rather heterogeneous and variable. The main motor characteristics are bradykinesia, muscle stiffness, tremor at rest, and postural and gait changes. Nonmotor characteristics include olfactory dysfunction, cognitive changes, psychiatric symptoms, sleep disorders, autonomic disorders, pain, and fatigue [1].

These various motor changes contribute to people with PD progressively decreased functioning—among which postural control changes stand out, impacting the activities of daily living (ADL), independence, and gait capacity [2, 3]. Postural instability is one of the signs of disease progression [4, 5] and a potential increase in the risk of falls [6–8], culminating in less social participation, greater sedentarism, and depression [9] and characterizing it as one of the greatest problems in PD [10]. Moreover, evidence indicates that the necessary use of dopamine in PD improves gait-related mobility without improving reactive postural responses, leading to a likely iatrogenic increase in the risk of falls, which would only be improved with rehabilitation and physical exercise [11].

Postural control may be expressed by measures such as the relationship between the support base and the center of mass, which has reflections on most functional motor tasks in humans [12, 13]. In PD, progressively impaired reactive and anticipatory postural control causes stability loss and both static and dynamic balance deficit, limiting gait and performance in routine activities, decreasing mobility, and possibly leading to falls. Consequently, a cycle of immobility and falls appears and is aggravated by the fear of falls due to postural instability [14, 15].

Physical exercises are a treatment option that may help breaking the cycle of immobility and postural instability [16]. The multimodal/multicomponent interventions are an option that simultaneously combining various forms of exercises to address different problems [17, 18]. They can enhance drug treatment effectiveness and delay disease progression, with long-term beneficial effects on motor aspects, routine activities, and the severity of PD [19].

Recent literature shows evidence that balance training can improve motor symptoms, mobility, balance confidence, and thus quality of life of people with PD [16, 20]. Osborne et al. [20] mention that balance training in aquatic environment can be considered over land-based therapy to improve fear of falling and quality of life. The use of different environments brings further neurosensory motor stimulus that are important for the motor skills [21, 22]. In the aquatic environment, these stimuli come from the physiological action of thermal and physical properties and also promote changes on the mechanics of the immersed body [23, 24]. Instability while immersed causes continuous postural adjustments, which also leads to proprioception and muscle control [25]. A study comparing aquatic and land environments found a reduction of fall rate in the hydrotherapy group [26]. The authors concluded that hydrotherapy allows a proprioceptive training that contributes to increase the limit of stability, giving the time to activate postural reactions to perturbations in protected conditions [26]. Thus, it can be understood that exercise in both environments can be additional and beneficial for persons with DP.

Hence, multimodal aquatic- and land-based interventions aiming for improved postural motor control outcomes are clinically relevant, as they may lead to functional maintenance or gains in people with PD. Thus, multimodal training has good initial evidence for PD—although systematic reviews and meta-analyses [19, 27, 28] point out gaps regarding the optimal dosage and effects of multimodal intervention programs (MIPs).

Therefore, the objective of this study was to verify the influence of a sequential MIP in aquatic (AES) and land environments (LES) on balance, postural control, motor aspects, and ADL in people with PD.

## 2. Materials and Methods

This is an interventional clinical study, addressing conditions before and after sequential multimodal interventions, as subjects participated in MIP in both AE and LE.

The study was registered in the Brazilian Registry of Clinical Trials under number RBR-6hnqcv and was approved by the Ethics Committee of the Department of Health Sciences of the Federal University of Paraná, Paraná, Brazil (CAAE 66781417.4.0000.0102 and Evaluation Report 2.200.372). The study followed the Transparent Reporting of Evaluations with Nonrandomized Design (TREND) statement checklist.

### 2.1. Participants

Recruiting, assignment, evaluation, and intervention took place from March 2018 to December 2019. Each intervention group had a maximum of 6 persons at the same appointment. People with a clinical diagnosis of idiopathic PD in stages 1 to 4 on the modified Hoehn and Yahr (H&Y) scale, of both sexes, aged more than 40 years, with a medical certificate to do physical activities, and attend a heated pool were included in the study by convenience. The exclusion criteria were as follows: not being able to walk, whether or not due to PD; having other diseases that might interfere with physical assessments (e.g., balance changes of vestibular origin); having visual or auditory sensory deficits that hindered participants from following verbal or visual instructions; being contraindicated to attend heated pools; changing levodopa-based drug intake parameters during the study period; changing baseline physical activities during the study period; not agreeing with the informed consent form; and missing assessments or more than 10% of interventions.

### 2.2. Assessment Procedures

Data were collected in the city of Curitiba, Paraná, Brazil, in partnership with the Parkinson's Association of Paraná.

The assessment and intervention were performed by physiotherapists with research and clinical experience in PD. All assessment scales were selected based on the European Guidelines for PD [29], in accordance with the domains in the International Classification of Functioning (ICF) [30] regarding PD. Participants were assessed in the “on” phase of the medication. Primary outcome was postural control. It was measured with Berg Balance Scale (BBS), Mini-Balance Evaluation Systems Test (Mini-BESTest), and postural control during quiet standing (QS) on a force platform. The secondary outcomes were motor aspects and ADL. They were assessed with Unified Parkinson's Disease Rating Scale (UPDRS) sections II and III and Dynamic Gait Index (DGI).

BBS was applied to assess the limited performance of ADL that requires static balance. It has 14 items on functional tasks, scoring 0 to 4 each, with a total score of 56—the higher the score, the higher the function level [31]. This tool helps establish the risk of falls in people with PD [29, 32], at the 52-point cutoff score for nonfallers and 47.6 for fallers [33].

Static and dynamic balance was assessed with the Mini-BESTest, which assesses the level of balance ability with 14 tests, scoring 0 to 2, with 4 subscores, anticipatory postural adjustments (6 points), reactive postural responses (6 points), sensory orientation (6 points), and gait stability (10 points). The maximum total score is 28 points; higher values indicate less impaired body balance [34, 35].

The UPDRS has 4 parts, of which parts II and III were used in this study. They, respectively, address motor experiences of daily living and motor functions and activities. The score ranges from 0 (normal) to 4 (severe)—higher scores are related to greater impairments [29, 36].

Balance during gait activities was assessed with DGI [37], which comprises eight activities that reflect the capacity to change gait according to task requirements in the scale. Scores range from 0 to 3 in each item—0 indicates the lowest functioning level and the greatest likelihood of falls. The maximum total score is 24, whereas scores equal to or lower than 19 indicate a greater risk of falls [38].

The QS assessment aimed to identify how the support base positioning and visual afference influence postural control by quantifying center of pressure (COP) sway before and after the interventions. Participants stood still on a force platform (AMTI^®^, model OR-06, USA) with 100 Hz sampling frequency, upper limbs parallel to the trunk, three times, for 30 seconds in each foot posture, in two conditions: (i) feet together and eyes open (EO_FT); (ii) feet together and eyes closed (EC_FT). In the eyes-open task, participants looked at a fixed point at eye level, 2 meters away [34]. In the first assessment, the positioning of each participant's feet was drawn on a paper the size of the platform, which was used in the subsequent assessments. The total length of COP trajectory (TL), anteroposterior (APA) and mediolateral COP amplitude (MLA), total sway displacement (TSD), total mean velocity (TMV), and area (AREA) of COP displacement was obtained and analyzed following the routine described by Lazarotto et al. [39], in MatLab^®^ 9.1.0 (MathWorks, Inc.).

**Figure 1:**
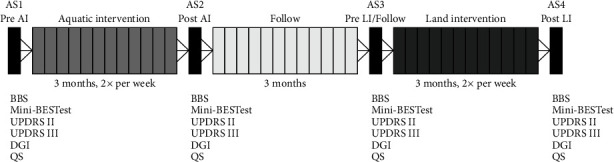
Overview of assessments and interventions. BBS: Berg balance scale; Mini-BEST: mini-balance evaluation system test; UPDRS: unified Parkinson's disease rating scale; II: activities of daily living; III: motor aspects; DGI: dynamic gait index; QS: quiet stand; AS: assessment; AI: aquatic intervention; LI: land-based intervention.

Assessments took place in four moments (Figure 1), namely: AS1-initial assessment, before the aquatic intervention; AS2-reassessment, after 12 weeks of aquatic intervention; AS3-after a 12-week follow-up with no interventions, which was also used as assessment before the land-based intervention; AS4-final reassessment after 12 weeks of land-based intervention.

The effects of the interventions were verified by comparing aquatic intervention to baseline (AS2-AS1), follow-up to identify retention of results 3 months after aquatic intervention (AS3-AS2) and to identify retention of results in relation to before any intervention (AS3-AS1). After land-based intervention, it was compared with the state of participants before it (AS4-AS3) and full intervention (aquatic+land-based) (AS4-AS1).

### 2.3. Intervention Program

The MIP was conducted sequentially in AE and LE for 12 weeks, twice a week, with 1 hour sessions, and a 12-week interval (follow-up) between them. It was based on the European Physiotherapy Guideline for PD [29], following the premise of oriented task training for gait, lower limb muscle strength and power, and static and dynamic balance.

MIP had the same structure for both environments, comprising three phases: 1: warm-up (15 minutes); 2: the main part (30 minutes); and 3: relaxation/cooldown (5 minutes) (Table 1). The complete description of aquatic intervention (AI) can be found at Siega et al. [40]. The land-based intervention (LI) exercise program is described at Iucksch [41]. The interventions took place at Rehabilitation Hospital of Paraná in Curitiba, Paraná.

The interventions had load progressions every 4 weeks. Functional mobility activities progressed as they were asked to pay greater attention, use higher quality movements, and use greater velocity and step length in gait exercises. Muscle strength and power training progressed in intensity according to the Borg rating of perceived exertion scale [42], gradually increasing every 4 weeks and maintaining the exertion level between 12 and 17 points. In AE, load progressed individually and every 4 weeks by including hydrodynamic equipment (swim fin), and in LE, by the resistance of elastic bands. Regarding balance exercises, new exercises were included every 4 weeks, with increasing complexity, less support of upper limbs, and a smaller support base.

### 2.4. Statistical Analysis

The comparison analysis between the various moments (AS1, AS2, AS3, and AS4) in the same group was made with Friedman ANOVA (*χ*2) and multiple-paired comparisons with Wilcoxon signed-rank (*z*) and Bonferroni correction, in SPSS^®^ Statistics 27. The effect size was calculated by *r* (*r* = *z*/*n*).

## 3. Results

The study sample comprised 18 people with PD (66.83 ± 11.74 years; 95% CI 60.99-72.67), most of them (66.7%) males, in H&Y stages 1 to 4, median 3; stages 2 and 4 were the most frequent ones in the sample (Table 2).

AI and LI had positive effects on static and dynamic balance, motor aspects, and ADL in people with PD, assessed with BBS, Mini-BESTest, and UPDRS II and III (Table 3). BBS results were significant (*χ*2[3] = 22.732, *p* < 0.001), with improved balance after AI (*z* = −3.394, *p* = 0.004; *r* = −0.88) and a large effect size—which were maintained in the follow-up (*z* = 2.404, *p* = 0.097) and in the full intervention (FI) that represents AI combined with LI (*z* = −3.536, *p* = 0.002; *r* = −0.91), with a large effect size.

The difference in the Mini-BESTest (*χ*2[3] = 19.305, *p* < 0.001) was in improved balance after LI (*z* = −3.323, *p* = 0.005; *r* = −0.86) and FI (*z* = −3.748, *p* = 0.001; *r* = −0.97), both with a large effect.

The proposed MIP significantly improved the ADL, assessed with UPDRS II (*χ*2[3] = 9.794, *p* = 0.020); UPDRS II scores were lower in the post-LI comparison (*z* = 2.687, *p* = 0.043; *r* = −0.69), with a medium effect.

Also, UPDRS III identified a significant motor improvement after AE and LE training (*χ*2[3] = 24.063, *p* < 0.001), with a trend toward lower scores after AI (*z* = 2.616, *p* = 0.053; *r* = 0.68), with a medium effect; after LI (*z* = 3.899, *p* = 0.001; *r* = 1.0), with a large effect; and in the end of the FI (*z* = 3.889, *p* = 0.001; *r* = 1.0), with a large effect. A trend toward losses was identified in the follow-up (between the end of AI and beginning of LI), with increased UPDRS III scores and medium effect (*z* = −2.616, *p* = 0.053; *r* = −0.68).

Balance and gait analyses with DGI found significance with Friedman ANOVA (*χ*2[3] = 8.290, *p* = 0.040). However, there were no differences in the comparisons; the median indicated a ceiling effect at the end of FI.

Postural control analysis with QS (TSD, TMV, APA, MLA, and AREA, in both conditions: EO_FT and EC_FT) identified no differences (Table 4) after AI and LI (*p* > 0.05).

**Figure 2:**
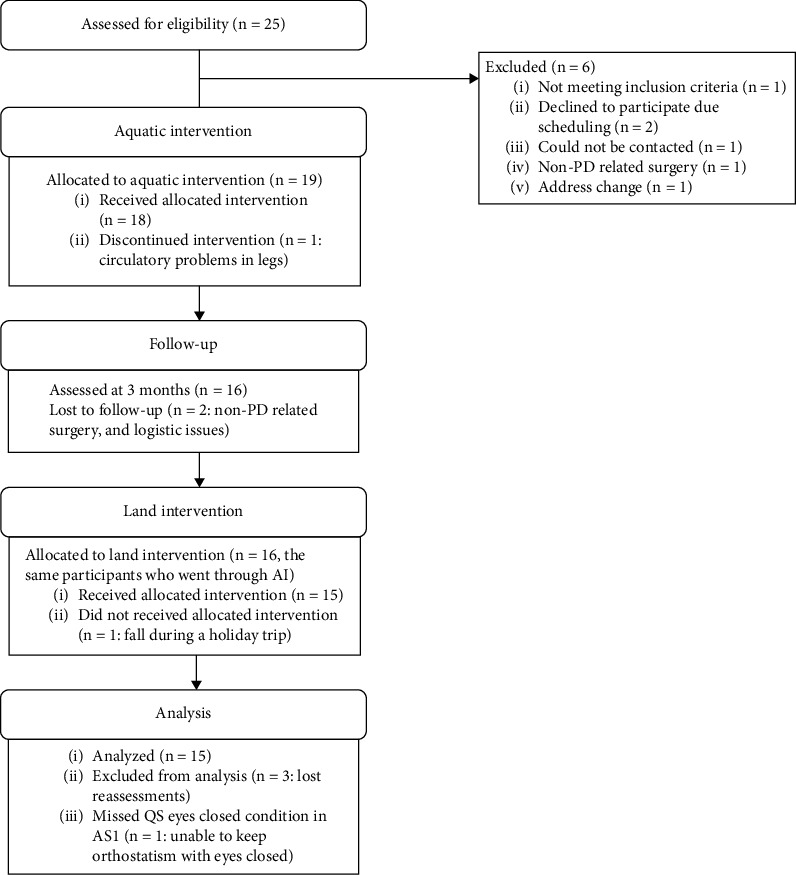
Participant flow chart.

One out of the 18 participants was unable to complete all QS conditions in AS1, whereas all of them were completed in AS2-AS4, indicating this participant's postural control improvement. Three people missed the reassessments, which is why the number of participants varied in the reassessments (Figure 2).

## 4. Discussion

This study verified that the proposed MIP in AE and LE improved static and dynamic balance, motor aspects, and ADL in people with PD. Postural control differences were verified with the functional scales (though not with QS variables), which justifies the use of complementary measure methods to assess such patients [29].

Although the sample comprised H&Y stages 1 to 4, most participants were in stages 2 and 4. Even in earlier stages, participants had balance and postural control changes, which justify early interventions to delay the deleterious effects of disease progression [19]. The differential of this study was the inclusion of people in H&Y stage 4 [43], confirming the positive effects of MIP even in people in more advanced PD stages, who are consequently more impaired.

BBS identified positive effects of MIP on the static balance after AI, with no significant losses in the follow-up. Masiero et al. [22] also found positive effects on balance with BBS after AI in people with PD H&Y stage 1.5 ± 0.5, in the “off” phase of the medication, after 4 weeks of exercises, twice a week. In our findings, the gains perceived with BBS were also verified in the FI, with a ceiling effect for some patients. This may have limited the verification of LE effects, as there were no losses in the follow-up, and they began LI with the effects transferred from AI.

Positive effects on balance verified with BBS were also reported by Volpe et al. [26]. They compared 34 people with PD, who received AE and LE intervention—with greater improvements in the AE. However, unlike those authors (who found an improved center of mass sway with eyes closed as well), no significant changes were identified in the postural control QS measures in the present study. Cugusi et al. [44], in a systematic review with meta-analysis, found more promising effects in AI than LI in BBS balance analysis—which, such as in the present study, were maintained in follow-up assessments.

Significant differences were identified with Mini-BESTest median values both after LI and in FI, with a 4-point variation. The minimal detectable clinical change in this test for H&Y stages 1 to 3 is 3.4 to 4.0 points in the group analysis [45]. Hence, LI and FI had positive effects even including patients in H&Y stage 4. This is relevant because PD people in H&Y stage 4 may have difficulties in 50% or more of the activities measured with Mini-BESTest [45] and therefore need opportunities to join exercise programs. The Mini-BESTest assesses balance domains with anticipatory adjustment, reactive postural control, sensory orientation, and dynamic gait [46]. MIP addressed these dimensions in both AI and LI.

The results of this study seemingly indicate that the AI phase of MIP influences more static balance components (measured with BBS), while LI influences more dynamic balance components (measured with Mini-BESTest). This may be explained by the evolution of components of postural control development—i.e., that static balance is firstly necessary so dynamic balance can be then improved [47]. This conclusion is reinforced by the fact that BBS reached a ceiling effect after AI while the Mini-BESTest result continued progressing until the end of the exercise program.

This study began the intervention in AE because of its hydrostatic and hydrodynamic properties and the safety they provide regarding the risk of falls, especially in more severe H&Y stages. The immersed body is knowingly unstable, requiring continuous postural adjustments, which further explain the balance improvement results after AI [48], with consequences in LI [25]. In AI, the center of body mass changes, requiring stabilization between gravity, density, and buoyancy forces, which in turn may cause rotational movements until finding balance, called metacentric effects [49]. Adequate postural balance must be maintained to stabilize the forces that act upon the immersed body. Therefore, aquatic activities can be a good alternative to begin postural control, with the additional benefit of providing safety to patients and therapists. Hence, the challenging activities and external disturbances make the development and training of motor skills easier [25].

The positive consequences on motor aspects and ADL obtained with UPDRS II and III demonstrate that the interventions were relevant to transferring the effects achieved in AE to ADL, through LI, thus justifying the complementary different therapy modalities. After LI, people with PD performed better in UPDRS II and III, and FI had positive effects on UPDRS III.

In UPDRS II, Schrag et al. [50] suggest a 2-point clinical difference for H&Y stages 1 and 2 and a 3-point difference for H&Y stage 3. In the present study, as the data were nonparametric, comparisons were made with the medians, significantly differing 5 points after LI, even including people with more severe PD, in H&Y stage 4. Thus, important clinical effects for the ADL of participants with PD were demonstrated and confirmed with the medium effect size obtained.

As for UPDRS III, the clinical difference is 5 points for people in H&Y stages 1 to 3 [50]. In this study, the medians significantly differed 3 points after AI and 4 points after LI and in the combination of the two environments. The effect size was greater after LI and FI than after AI alone. These findings may be related to the time of intervention; in this case, the cumulative effect of interventions and time would be necessary to verify the results in motor aspects. They may also be related to the similarity with the environment where UPDRS tasks are assessed.

Since people with more severe PD (H&Y stage 4) were included in the study, these values and the nonnormal distribution may have influenced the identification of results. Therefore, studies with a larger and more representative sample for each PD stage should be conducted, even though they pose a challenge to clinical research.

The MIP had specific exercises for lower limb balance and strength in both environments. As the intensity progressed, patients were always encouraged to maintain the quality of movement, full attention, and cognition. From a clinical standpoint, Horak [51] states that postural control efficiency depends on six main points: biomechanical restrictions, movement strategies, sensory strategies, space orientation, dynamic control, and cognitive processes. Thus, reinforcing the complex interaction between multiple sensory-cognitive-motor processes [47, 52] and the need for a variety of progressive stimuli in different environments.

In DGI analysis, the final median of the MIP was 24—the maximum value with ceiling effect—which indicates the participants' improvement, though making it difficult to find where the differences in statistical analyses were. Although DGI is included in the PD recommendation guidelines, other instruments, such as the Functional Gait Assessment (FGA), may be more sensitive to detecting postintervention changes [29].

The QS analysis in this study did not identify any difference in postural control after MIP. Despite the advancements, the complex physiopathology of postural instability in PD has not been fully clarified yet because of the difficulty distinguishing the disease process and the patients' compensatory mechanisms, besides the lack of standardized measuring techniques [53]. QS analysis with a force platform is a quantitative alternative to obtain data on postural stability in PD. However, the results in this study did not follow the other instruments used. An explanation may be the sample size, which made it possible to find changes on scales with discrete values, but not in continuous variables such as the ones acquired in the force platform. Another possibility is that the QS analysis verified sway aspects without reflecting the functional characteristics, whose outcomes are measured with clinical scales that allow an application more related to everyday life experience.

The limitations of the study and suggestions for future research include having a control group, different intervention sequence combinations, and greater sample size to allow separate analysis by H&Y stages.

## 5. Conclusions

This research identified improved balance, ADL, and motor aspects in people with PD after an MIP was applied sequentially in AE and LE. The combination of aquatic- and land-based interventions seems to be complementary in the treatment of people with PD. It is understood that physical exercises, regardless of the environment where they are done, bring benefits to people with PD, breaking the cycle of limited mobility and improving their functioning and quality of life.

## Figures and Tables

**Table 1:** Multimodal intervention program framework.

Warm-up (15 minutes)	Main part (30 minutes)	Cooldown (5 minutes)
Functional mobility exercises: (i) Gait in different directions and velocities, with turns, squats, pelvic, and scapular girdles (ii) Cross the training area with hops and runs	(a) Lower limb muscle strength and power (i) Kicking with the right and left leg (ii) Abducting/adducting right and left leg (b) Balance exercises (i) Postural shift (ii) Reduced base exercises (iii) Gait with floating equipment (which increased instability) and obstacles (iv) Maintain orthostatism on the aquatic trampoline with external perturbation	Initial Ai Chi postures in aquatic environment Initial tai chi postures in land environment

**Table 2:** Sample characterization.

*n* = 18	Mean ± SD	95% CI
Age (years)	66.83 ± 11.74	60.99-72.67
	*n*	%
Sex^∗^	*M* = 12	66.7
	*F* = 6	33.3
Hoehn & Yahr^∗^		
1	1	5.6
1.5	1	5.6
2	6	33.3
3	4	22.2
4	6	33.3
Median (interquartile range)	3	(2-04)

SD: standard deviation; CI: confidence interval; M: males; F: females. ^∗^Shapiro Wilk; *p* < 0.05.

**Table 3:** Comparative analysis of balance, motor aspects, and activities of daily living of people with Parkinson's disease after the sequential multimodal intervention program in aquatic and land environments.

Environment	AE		LE		*χ*2 (df)	AE		LE			
Scales	AS1	AS2	AS3	AS4		AS2-AS1	AS3-AS2	AS3-AS1	AS4-AS3	AS4-AS1	
	Median										
*n* = 15	Interquartile range (25-75%)				*p*	AI	Follow-up		LI	FI	
BBS	51	53	50	54	22.732 (3)	-3.394	2.404	-0.990	-2.546	-3.536	**z**
	(44-55)	(49-56)	(45-55)	(51-56)	**<0.001**	**0.004**	0.097	1.000	0.065	**0.002**	**p**
						-0.88	—	—	—	-0.91	**r**

Mini-BEST	22	23	22	26	19.305 (3)	-1.485	1.061	-0.424	-3.323	-3.748	**z**
	(18-26)	(20-27)	(15-26)	(22-27)	**<0.001**	0.825	1.000	1.000	**0.005**	**0.001**	**p**
						—	—	—	-0.86	-0.97	**r**

UPDRS II	14	11	15	10	9.794 (3)	1.273	-2.121	-0.849	2.687	1.838	**z**
	(9-15)	(8-13)	(8-17)	(7-16)	**0.02**	1.000	0.203	1.000	**0.043**	0.396	**p**
						—	—	—	0.69	—	**r**

UPDRS III	15	12	15	11	24.063 (3)	2.616	-2.616	0	3.889	3.889	**z**
	(10-17)	(8-15)	(10-19)	(8-13)	**<0.001**	0.053	0.053	1.000	**0.001**	**0.001**	**p**
						0.68	-0.68	—	1.000	1.000	**r**

DGI	21	23	23	24	8.290 (3)	-0.566	0.354	-0.212	-1.838	-2.051	**z**
	(19-24)	(21-24)	(19-24)	(24-24)	**0.04**	1.000	1.000	1.000	0.396	0.242	**p**
						—	—	—	—	—	**r**

BBS: Berg balance scale; Mini-BEST: mini-balance evaluation system test; UPDRS: unified Parkinson's disease rating scale; II: activities of daily living; III: motor aspects; DGI: dynamic gait index; AS: assessment; AE: aquatic environment; LE: land environment; AI: aquatic intervention; LI: land-based intervention; FI: full intervention. *χ*2 (df): Friedman ANOVA (degrees of freedom); *z*: multiple comparisons with Wilcoxon signed-rank and Bonferroni correction, *p* < 0.05; *r*: effect size (*r* = *z*/√*n*).

**Table 4:** Comparative analysis of postural control in a still upright posture in people with Parkinson's disease after the sequential multimodal intervention program in aquatic and land environments.

Environment			AE		LE		
Still upright posture		*n*	AS1	AS2	AS3	AS4	
			Median interquartile range (5-75%)				**χ**2**(df)**
							**p**
EO_FT	TSD	15	22.58	21.1	21.19	20.86	3.560 (3)
			(13.55-30.86)	(17.09-25.16)	(11.31-26.19)	(15.21-24.46)	0.313
	TMV	15	1.69	1.75	1.68	1.82	1.080 (3)
			(1.41-2.38)	(1.40-2.29)	(1.21-2.79)	(1.42-2.51)	0.782
	APA	15	2.91	2.81	2.51	2.48	2.120 (3)
			(1.93-3.39)	(1.59-3.08)	(1.53-2.96)	(1.73-2.95)	0.548
	MLA	15	3.53	2.91	2.7	2.99	5.560 (3)
			(1.95-4.49)	(2.45-3.68)	(1.68-3.81)	(2.08-2.99)	0.135
	AREA	15	6.13	5.52	5.54	5.71	2.760 (3)
			(2.53-11.52)	(3.28-8.05)	(1.63-8.66)	(2.23-7.54)	0.43

EC_FT	TSD	14	25.59	21.95	23.33	23.98	2.829 (3)
			(18.95-29.8)	(16.84-33.58)	(15.06-28.14)	(15.69-25.67)	0.419
	TMV	14	2.17	2.48	2.47	2.47	0.086 (3)
			(1.70-3.54)	(1.68-3.68)	(1.69-3.52)	(2.12-2.95)	0.993
	APA	14	3.22	3.17	2.81	2.95	4.543 (3)
			(2.47-3.75)	(1.95-3.64)	(2.34-3.67)	(2.13-3.7)	0.208
	MLA	14	3.84	3.5	3.55	3.73	0.943 (3)
			(2.59-4.55)	(2.53-5.19)	(2.27-4.46)	(2.49-4.28)	0.815
	AREA	14	8.65	6.71	6.92	7.47	1.886 (3)
			(4.71-10.5)	(3.9-13.7)	(2.66-10.11)	(3.28-8.53)	0.596

TSD: total sway displacement; TMV: total mean velocity; APA: anteroposterior amplitude; MLA: mediolateral amplitude; AREA: sway area; EO: eyes open; EC: eyes closed; FT: feet together; AS: assessment; AE: aquatic environment; LE: land environment. *χ*2 (df): Friedman ANOVA (degrees of freedom), *p* < 0.05.

## Data Availability

Research data cannot be shared due to ethical issues regarding their secrecy and nondisclosure according to the Research Ethics Committee of the Federal University of Paraná (UFPR), Brazil. Only the study results may be shared.
